# Comparative psychopharmacology of autism and psychotic-affective disorders suggests new targets for treatment

**DOI:** 10.1093/emph/eoz022

**Published:** 2019-08-26

**Authors:** Bernard J Crespi

**Affiliations:** Department of Biological Sciences, Simon Fraser University, 8888 University Drive, Burnaby, BC V5A 1S6, Canada

**Keywords:** autism, schizophrenia, psychopharmacology, evolution, psychosis

## Abstract

The first treatments showing effectiveness for some psychiatric disorders, such as lithium for bipolar disorder and chlorpromazine for schizophrenia, were discovered by accident. Currently, psychiatric drug design is seen as a scientific enterprise, limited though it remains by the complexity of brain development and function. Relatively few novel and effective drugs have, however, been developed for many years. The purpose of this article is to demonstrate how evolutionary biology can provide a useful framework for psychiatric drug development. The framework is based on a diametrical nature of autism, compared with psychotic-affective disorders (mainly schizophrenia, bipolar disorder and depression). This paradigm follows from two inferences: (i) risks and phenotypes of human psychiatric disorders derive from phenotypes that have evolved along the human lineage and (ii) biological variation is bidirectional (e.g. higher vs lower, faster vs slower, etc.), such that dysregulation of psychological traits varies in two opposite ways. In this context, the author review the evidence salient to the hypothesis that autism and psychotic-affective disorders represent diametrical disorders in terms of current, proposed and potential psychopharmacological treatments. Studies of brain-derived neurotrophic factor, the PI3K pathway, the NMDA receptor, kynurenic acid metabolism, agmatine metabolism, levels of the endocannabinoid anandamide, antidepressants, anticonvulsants, antipsychotics, and other treatments, demonstrate evidence of diametric effects in autism spectrum disorders and phenotypes compared with psychotic-affective disorders and phenotypes. These findings yield insights into treatment mechanisms and the development of new pharmacological therapies, as well as providing an explanation for the longstanding puzzle of antagonism between epilepsy and psychosis.

Lay Summary: Consideration of autism and schizophrenia as caused by opposite alterations to brain development and function leads to novel suggestions for pharmacological treatments.

## INTRODUCTION

The development of effective pharmacological treatments for human psychiatric and neurological disorders represents one of the most challenging fields of biology, due mainly to current limitations on our understanding of neurodevelopment, neurological function and the links of neuroscience with psychiatry. In seeking to develop new drugs, psychopharmacologists must draw upon knowledge from a broad and deep range of disciplines, from genetics to biochemistry, neurophysiology, neuroanatomy and neuropsychiatry. The majority of new treatments nonetheless fail in early clinical stages, for reasons that commonly remain unknown, and most new drugs, such as lithium for bipolar disorder, have been discovered by accident (e.g. [1]). The only relevant field missing, apparently, from the intellectual toolkit of psychopharmacology, is evolutionary biology. How might this discipline, which unites all of the life sciences, be useful in such a context?

The purpose of this article is to describe and evaluate a comparative evolutionary framework for pharmacological treatment of autism spectrum and psychotic-affective spectrum disorders. The framework is based on Crespi and Badcock’s [2] theory that these two sets of disorders are generally opposite (diametrical) to one another, in that autism is a syndrome mediated by underdevelopment of the highly human-evolved social brain (and overdeveloped nonsocial traits including aspects of perception, attention and intelligence), while psychotic-affective spectrum disorders, comprising mainly schizophrenia, bipolar disorder, depression, borderline personality and related conditions, are mediated by forms of maladaptive overdevelopment of the same set of social phenotypes (and underdeveloped nonsocial ones). Autism is regarded as a heterogeneous syndrome (a constellation of physiological, neurological and psychological phenotypes, sets of which are commonly found together in a given individual) [3]. Psychotic-affective conditions are a set of disorders that partially share phenotypes (including the presence of psychosis, and typically, mood disorder) as well as being strongly correlated genetically and thus overlapping in their genetic underpinnings; these disorders have discrete names but each represents a more or less heterogeneous syndrome that grades into one or more of the related disorders [4–8].

Genetic, morphological, neurological, psychological and other evidence relevant to the diametric model is most-recently summarized in Crespi and Go (Table 2 in [9]), Crespi [10, 11], Crespi *et al.* [12] and Dinsdale *et al.* [13]. The diametric structure of this model is not unique to psychology and psychiatry: at least four additional sets of human disorders show clear evidence of diametric etiologies, including osteoarthritis versus osteoporosis, cancer versus neurodegeneration and senescence, infectious versus autoimmune disease risks, and anorexia versus obesity [3, 14–16].

**Figure 1.**
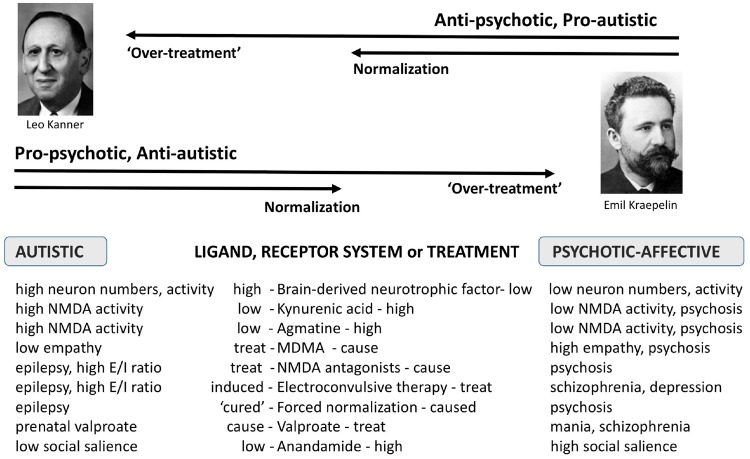
Model of autism and psychotic-affective conditions as diametric (opposite) disorders with diametric treatments and psychopharmacological patterns related to treatments. Optimal levels of treatment lead to normal, balanced cognition, while ‘too much’ treatment leads to phenotypes typical of the opposite disorder or disorders. See text for details. Leo Kanner is known for describing autism, and Emil Kraepelin for describing schizophrenia

Application of the diametric model to the psychopharmacologies of autism and psychotic-affective disorders is predicated on the fact that if the neurophysiological and neurochemical causes and correlates of these disorders are broadly opposite to one another, then their treatments should be as well. Robust evaluation of the hypothesis that autism and psychotic-affective disorders represent opposites, as regards pharmacological treatments, requires explicit predictions and tests. The primary predictions addressed here are 4-fold:

1. Autism and psychotic-affective disorders such as schizophrenia are expected to show opposite alterations to levels of neurochemicals and pathway activity that mediate their causes and symptoms, higher than typical in one disorder, and lower in the other.
2. Antipsychotic, mood stabilizer and antidepressant drugs or other treatments should tend to direct neurodevelopment and function in the ‘autistic’ direction, toward normality and then, if ‘over-corrected’, toward autism-related traits and autism risk (Fig. 1). The effects of such shifts depend upon the magnitude of the perturbation, and the psychological state or ‘starting-point’ of the individual on a continuum from autism to normality to the relevant psychotic-affective condition.
3. Propsychotic and prodepressant drugs or treatments should tend to decrease autism risk and autism-related phenotypes, in parallel to the points outlined in (1) above.
4. Strong correlates of autism, such as epilepsy and highly focused attention, should represent or indicate foci for treatments for psychotic-affective disorders, and vice versa for strong correlates of psychotic-affective disorders.

The rationale and a summary of the evidence regarding these predictions, in the context of the diametrical model [2, 9, 10], is illustrated in Fig. 1.

These predictions are subject to a number of important caveats and other considerations. First, autism, schizophrenia, depression, bipolar disorder, borderline personality disorder and psychosis, are each highly heterogeneous disorders as regards their specific proximate causes [10]. As such, each of the disorders has many possible etiologies at the genetic and environmental levels, which converge in each case to a much smaller set of characteristic neurophysiological, neurodevelopmental, neuroanatomical, cognitive, psychological and finally psychiatrically diagnostic traits. A primary consequence of this causal diversity and psychological-behavioral convergence is that a DSM-based diagnosis of, say, autism or schizophrenia should be considered as the start of the main, ‘true’ differentially diagnostic process, to determine the personalized genetic and environmental causes of the disorder for each individual. These personalized causes will determine the appropriate pharmacological treatment (if any) to deploy in each case. Notwithstanding these complexities, the set of treatments used for autism spectrum disorders should be broadly diametric, as regards receptor and pathway agonistic or antagonistic effects, to the treatments used in psychotic-affective disorders.

Second, psychiatric diagnoses, such as those based on the DSM, may be subject to certain patterns and degrees of mistakes. For autism and psychotic-affective disorders, specific sorts of mistakes are expected based on the fact that autism spectrum disorders are overwhelmingly diagnosed in childhood, while psychotic-affective disorders are typically diagnosed in adolescence or adulthood yet often manifest in social, psychological and other deficits in childhood. As a result, children who are ‘premorbid’ for psychotic-affective conditions (i.e. are expected to develop them), especially those with relatively penetrant risk factors such as genomic copy-number variants, are expected to frequently be diagnosed in childhood with autism spectrum disorders. Such diagnoses can be considered as false positives [17], and they are observed, in the literature, either as diagnoses of autism in childhood and, e.g. schizophrenia in adulthood, or as associations of one copy-number variant (e.g. a deletion at 16p11.2) exclusively with autism, while its reciprocal variant (a duplication of the same 16p11.2 region) is associated with both schizophrenia and (as an apparent false positive), autism [18–20]. Evidence regarding premorbidity to schizophrenia diagnosed as autism spectrum disorders is described in more detail in related publications [18–20].

Adolescents or young adults with schizophrenia or bipolar disorder are expected to be much more rarely diagnosed incorrectly with autism spectrum disorders, since the positive symptoms of these two disorders, especially hallucinations, delusions and mania, are relatively specific. The upshot of this situation is that some proportion of children or young adolescents diagnosed with autism actually has a psychotic-affective condition that has yet to show its diagnostic-specific symptoms because the subject is too young. The resulting diagnostic asymmetry must be kept in mind when interpreting the literature. Similar, but more pronounced, considerations apply to the use of mouse and rat models for, especially, autism and schizophrenia, because the same social or task deficits can be interpreted as evidence for effects of each disorder. In contrast to such ambiguities, such animal models are highly useful for analyses of the proximate neurological causes of well-characterized genetic alterations linked with these disorders among humans.

Third, there are currently no approved, effective pharmacological treatments available for treating the core symptoms of idiopathic (cause-unknown) autism, although the atypical antipsychotics aripiprazole and risperidone are approved by the US FDA for alleviating irritability, aggression and self-injury in autism [21]. In contrast, treatments for some single-gene autistic syndromes, including, e.g. Rett, Angelman, Fragile X, TSC1 and TSC2, and others have been developed and tested and are discussed here.

Given these limitations, a set of phenotypes and symptoms that are closely associated with autism, including, e.g. reduced empathy [22], macrocephaly (large head size) [23], and high rates of epilepsy and epileptiform EEGs in association with high neural excitation to inhibition ratios [24–28], can serve as proxies for autism spectrum traits modifiable by pharmacological treatment. Details regarding these proxies, and any caveats that may apply to their use, are discussed in the relevant sections below. It is important to bear in mind that these and other phenotypic correlates of autism each applies in only a subset of cases, given the high heterogeneity of this disorder.

The predictions of the hypotheses addressed here are evaluated through discussion of the set of ligand, drug and receptor, pathway systems, and other forms of treatments and effects, for which there is sufficient information regarding effects on both autistic and psychotic-affective diagnoses and phenotypes. The review is in narrative form, given that data have not previously been collected in the context of the hypothesis under consideration.

## RESULTS

### Brain-derived neurotrophic factor

Brain-derived neurotrophic factor (BDNF), the most prevalent neurotrophic growth factor in the brain, mediates neurodevelopment and synaptic plasticity [29–31], with notable effects on learning and memory through downstream effects of activation of its receptor TrkB [32].

Levels of BDNF in serum and plasma are higher among subjects with autism than in matched controls, by four meta-analyses of overlapping datasets [33–36]. In contrast, BDNF levels are lower in schizophrenia [37], bipolar [38] and depression [39], also using meta-analyses in each case. BDNF levels are positively correlated with autism traits, by the Autism Quotient questionnaire, in a non-clinical population [40].

Additional evidence linking higher levels of BDNF with autism includes: (i) its role in promoting neuronal survival [41], given larger brain size and reduced neuronal pruning in autism; (ii) higher BDNF among individuals with Angelman syndrome, an autistic condition, than among controls [42] but lower levels in Prader−Willi syndrome, a penetrant cause of affective psychosis [43, 44]; (3) higher BDNF in fetal brain of mice prenatally exposed to valproic acid, a well-validated model of autism [45, 46]; and (4) links of high BDNF levels with epilepsy, involving both BDNF-induced seizures and higher BDNF after seizures [47–50], given the strong associations of epilepsy with autism [24–28, 51]. In contrast, schizophrenia is characterized by reduced neural synchrony [52–54], apparently in part due to the antiepileptic effects on NMDA receptor antagonism [55] epilepsy is primarily found in association with temporal lobe activation, especially involving the hippocampus, that specifically mediates hallucinations and delusions [56, 57].

Associations of low BDNF with psychotic-affective disorders are also supported by (i) a negative correlation of BDNF levels with psychotic symptoms of schizophrenia [58] and (ii) increased BDNF after treatment with antipsychotic and antidepressant drugs [38, 59, 60].

The BDNF-trkB system has been considered as a pharmacological target for both a hyperactive system in autism [61], and for underactivity in schizophrenia and depression [62, 63]. Direct comparisons of the BDNF-trkB system dynamics between autism and psychotic-affective disorders, which have yet to be conducted, should provide useful insights into the potential of this neurotrophin for therapeutic treatment.

### PI3K-Akt-mTOR pathway

**Figure 2.**
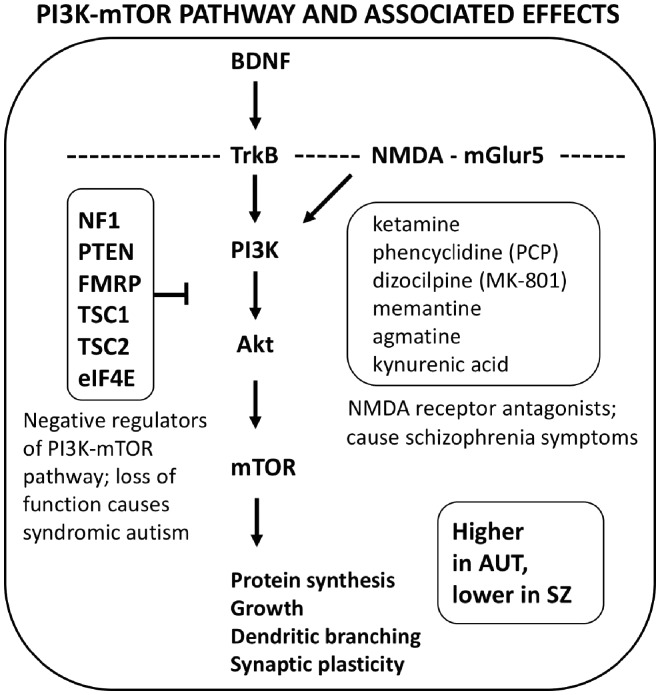
Highly simplified depiction of the inter-relationships of the BDNF-TrkB, NMDA-mGLur5, and PI3K- mTOR systems, that impact upon pathways highly relevant to autism and psychotic-affective conditions. Arrows refer to activation, and the blunt tip refers to negative regulation (reducing activation). NMDA antagonists cause psychological and physiological shifts in the direction of the phenotypes of psychosis. Dashed line refers to synaptic cell membrane. See text for details

The PI3K-Akt-mTOR pathway mediates intracellular signaling in control of the cell cycle across cell and tissue types; as such, it controls major aspects of brain and body growth, differentiation and cell turnover [64, 65]. In neurons, this pathway regulates growth, cell survival, protein synthesis, NMDA-dependent synaptic plasticity, and dendrite growth and branching, through activation by growth factors including BDNF, as well as through stimulation by NMDA and mGLUR5 receptors (Fig. 2). As such, this pathway, and the systems with which it interacts (especially the Wnt, MAPK and ERK pathways), play central roles in neurodevelopment and neuronal function, through increases versus decreases in activity as a whole and in its component parts.

Multiple lines of evidence indicate that the PI3K-Akt-mTOR pathway is overactivated in autism spectrum disorders. The most direct evidence comes from monogenic, syndromic forms of autism, many of which are caused by lost or reduced function in genes, including eIF4E, FMR1, PTEN, NF1, TSC1, TSC2 and PRKCB1, that negatively regulate the pathway or its subsystems [66–72] (Fig. 2). Indeed, Hoeffer and Klann [73] noted that ‘although single-gene sources account for only 8–15% of all ASDs, more than half are involved in direct regulation of either mTor signaling or translation control’. An important role for upregulation of the PI3K-Akt-mTOR in autism more generally is indicated by significant higher pathway activity in cells of individuals with idiopathic autism, than in matched controls [74]. High pathway activity has also been directly implicated in a suite of strong correlates of autism, including large brain size [75–77], epilepsy and high E/I ratios [78, 79], and increased dendritic growth and branching [80].

Neurodevelopmental and neurophysiological mechanisms whereby upregulated PI3K-Akt-mTOR may mediate autism spectrum disorders include excessive brain growth and neuron numbers, increased protein translation at synapses [81, 82], and high E/I ratios in key neural systems (e.g. [83]). Pharmacological treatments based on inhibition of this pathway have been developed and tested in mouse models for Fragile X syndrome [84], eIF4E dysregulation [85], and TSC1 and TSC2 [86, 87], with amelioration of autism-associated deficits in all cases. mTOR activity is also increased in the valproic acid rodent model of autism, with alleviation of symptoms by administration of the mTOR antagonist rapamycin [88].

Whereas autism is characterized by increased PI3K-Akt-mTOR activity, a substantial body of evidence indicates that this pathway is downregulated in schizophrenia [71, 89–93]. This evidence derives mainly from studies of the pathway-related effects of particular schizophrenia risk alleles and genes, analysis of mouse model knockouts out for pathway activators, and studies of the effects of schizophrenia treatments on pathway activation [71, 90, 92]. Notably, pharmacological treatment with antipsychotics leads to increased pathway activation in mouse and cell-based models of schizophrenia (e.g. [89, 94–99]), and in the latter study, the antipsychotic haloperidol also increased dendritic spine density in an mTOR-dependent manner. Electroconvulsive therapy animal models also show that this treatment increases PI3K-Akt-mTOR activation [100], suggesting that it contributes to the efficacy of this approach.

Finally, in contrast to the large brains, elevated protein translation at synapses, and high dendritic complexity in autism noted above, schizophrenia is characterized by small brain size [101, 102], decreased dendritic growth and branching [103] as well as reduced neuronal protein synthesis due to lower mTOR activation [104]. Schizophrenia thus shows an opposite pattern to autism with regard to PI3K-Akt-mTOR pathway activation. Studies are needed that directly compared autism and schizophrenia with regard to neuronal PI3K-Akt-mTOR activity and its effects on neuronal survival, protein synthesis levels and patterns, synaptic plasticity and dendritic spine phenotypes.

### Kynurenine pathway

**Figure 3.**
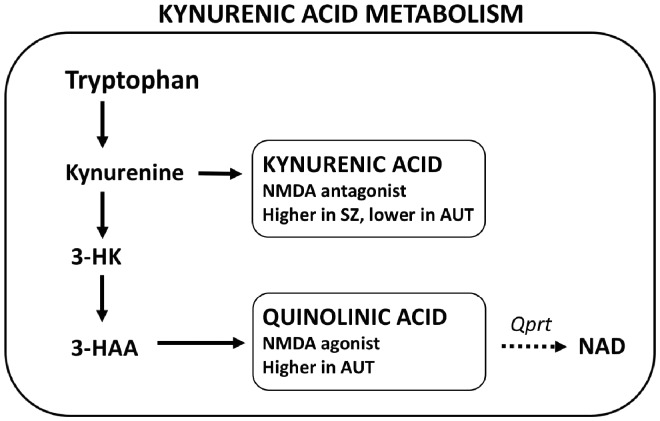
Simplified depiction of kynurenic acid pathway metabolism, which mediates risk and phenotypes of autism and schizophrenia via effects on NMDA receptor activity levels

The kynurenine pathway controls the enzymatic conversion to the amino acid tryptophan to two end products, kynurenic acid (KYNA) and quinolinic acid (QUIN), both of which are neuroactive (Fig. 3); this pathway also leads to the production of nicotinamide adenine dinucleotide (NAD+). Kynurenic acid functions as an antagonist of the NMDA receptor, with pro-psychotic neurophysiological and behavioral effects similar to those of other NMDA antagonists such as ketamine; it also antagonizes the a7 nicotinic acetylcholine (a7nACh) receptor, with high levels of antagonism leading to deficits in attention, learning, memory via modulation of synaptic plasticity [105]. QUIN, in contrast, acts as an agonist of the NMDA receptor, leading in relatively-high concentrations to seizures and excitotoxicity [106–108].

Variation in activity and products of the kynurenine pathway mediates both neurodevelopment and neuronal function, and has been analysed in schizophrenia, autism, and other neurological conditions [109, 110]. Concentrations of KYNA in cerebrospinal fluid are substantially increased among subjects with schizophrenia, across many studies ([111–114], meta-analysis in [115]); comparable increases are also found among subjects with bipolar disorder with (but not without) psychosis, and KYNA levels are associated directly with psychotic symptoms in both disorders [105]. Treatment with antipsychotics reduces levels on KYNA, from studies in rodents [116, 117].

Levels of the NMDA agonist QUIN did not differ between controls and subjects with schizophrenia in two studies of cerebrospinal fluid concentrations [118, 119], but QUIN levels were lower among subjects with schizophrenia in a third study that was based on immunoreactivity in the hippocampus [120]. The ratio of QUIN to KYNA, a metric of net alterations to the kynurenic acid pathway, was measured or inferred as lower in schizophrenia subjects than in controls by both Kegel *et al.* [119] and Gos *et al.* [120], which is indicative of NMDA antagonism overall. Diverse, convergent evidence thus indicates that psychosis is characterized by high absolute and relative levels of KYNA, as expected given its antagonist effects on the NMDA receptor. Inhibition or deletion of the enzyme KAT II, resulting in lower levels of KYNA, is associated with enhanced cognition and antipsychotic effects in studies of rodents [111, 121, 122]; this and other means of lowering levels of KYNA represent active areas of pharmacological research [105, 114].

Study of kynurenic acid metabolism in autism spectrum disorders has proceeded independently of that for schizophrenia. Across the three independent studies of KYNA levels conducted thus far, two reported significantly lower levels in subjects with autism than in controls [123, 124], and one reported no differences [125]. Levels of QUIN were significantly increased among autism subjects in two of the three studies that also measured this metabolite [123, 125], and were not different in Bryn *et al.* [124]. Ratios of QUIN to KYNA were thus much higher in autism for two studies [123, 125] but did not differ in a third study [124]. Data available so far thus provide evidence that levels of KYNA are decreased, while QUIN and QUIN-to-KYNA ratios are increased, in autism compared with controls; this is the opposite pattern to that seen among subjects with schizophrenia, as described above. Further analyses are needed, however, especially ones that jointly compare subjects with autism and schizophrenia (and psychosis) for levels of KYNA and QUIN.

An independent line of evidence concerning kynurenine pathway alterations in autism comes from analysis of a large genomic copy number variant deletion at 16p11.2 that includes the gene QRPT, which codes for the enzyme that metabolizes QUIN [126]. Mouse knockouts of this gene exhibit higher levels of QUIN and increased expression of some subunits of the NMDA receptor [127]. Among humans, deletions of 16p11.2 are strongly associated with autism and seizures [128–130], and individuals with deletions show lower QPRT expression, while those with duplications exhibit higher expression [131]. Haslinger *et al.* [126] suggest that these findings implicate high QUIN, and the gene QPRT, in autism spectrum disorders, based on alterations to cell phenotypes and expression of ASD-associated genes in a neuronal cell model, as well as the high QUIN levels in idiopathic autism. Studies of kynurenine pathway metabolism among individuals with duplications of 16p11.2, who harbor three copies of the QPRT gene, would be of particular interest given the association of this duplication with elevated rates of schizophrenia [132]. More generally, the relative simplicity of dietary and pharmacological modulation of this pathway should compel studies that test for associations of KYNA and QUIN concentrations with levels of autism-related phenotypes, to determine if and how individualized interventions based on kynurenine metabolism may be useful.

### NMDA receptor antagonist and agonism

The NMDA receptor functions as an ion channel in neurons, with key roles in synaptic plasticity, learning and memory. Antagonism of this receptor with sufficient concentrations of neurochemicals including ketamine, phencyclidine (PCP), dizocilpine (MK-801) and others (Fig. 2) causes expression of the major cognitive and neurophysiological symptoms of psychosis [133], as well as inducing other phenotypes including empathy, euphoria and dissociation [134]. As such, these agents represent key model pharmacological systems for the study of psychosis and schizophrenia. Additional convergent evidence of a central role for NMDA glutamate receptor hypofunction in psychotic disorders includes: (i) associations of schizophrenia risk genes with effects on this receptor, (ii) receptor subunit dysregulation in postmortem brain of schizophrenia subjects and in animal models, (iii) interactions of glutamatergic neurons with GABA-ergic and dopaminergic neurons in current models of receptor-system alterations causing psychosis and schizophrenia and (iv) NMDA receptor activation effects of some antipsychotic drugs (e.g. [135–137]). Direct agonism of the NMDA receptor is problematic as therapy due to the risks associated with overactivation and glutamatergic excitotoxicity, so most research designed to alleviate its hypofunction has focused on positive allosteric modulation (lowering of activation threshold without blockade) of the receptor, as well as on agonism of the mGlur5 receptor, with which it interacts, neurophysiologically, in a positive manner [138–140]. mGLur5 hypofunction is also characteristic of schizophrenia [141].

The diametrical model of autism and psychosis, applied to NMDA function, predicts that this receptor should commonly be hyperactivated in autism, and that its antagonism or negative allosteric modulation (increase of activation threshold without blockade) should tend to reduce autism symptoms. Evidence of NMDA receptor hyperactivation in autism comes from three main lines of evidence: studies of human subjects with monogenic, syndromic forms of autism, studies of idiopathic autism and studies of autism mouse models, mainly knockouts involving genes that impact glutamatergic activity.

A suite of monogenic forms of autism that symptomatically resemble Angelman and Rett syndromes has been described in the context of contextualizing differential diagnostics [142]. All seven of these disorders that have been investigated for links with glutamatergic neurotransmission and the NMDA receptor show some combination of higher receptor activation and high E/I ratios more broadly; all also show high rates of seizures and autism or autism spectrum traits (Table 1). For three of these syndromes (Rett, Pitt-Hopkins and MEF2C knockout), symptoms are also known to be ameliorated by NMDA antagonists, in humans, mouse models or both. These findings indicate that a notable proportion of syndromic autistic conditions involves relatively high NMDA activity, and that treatment with agents that act as propsychotics in typical conditions tend to normalize symptoms in some cases in autism. Increased mGlur5 receptor activity has also been implicated in autism, most clearly for Fragile X syndrome [173]. Treatment of mouse fragile X models with mGlur5 receptor antagonists or negative allosteric modulators have led to notable symptom reductions (e.g. [174–176]); however, such benefits have not translated as yet to human trials, for reasons that remain unclear [177, 178]. As expected under the diametric model of autism and psychosis in psychopharmacology, some treatments with mGlur5 antagonists in autistic conditions result not just in tendencies toward normalization, but also in symptoms of psychosis (e.g. [179]) that are indicative of ‘over-effectiveness’.

**Table 1.** A set of neurogenetic disorders that resemble Angelman syndrome show a coincidence of NMDA receptor hyperactivity, high rates of epilepsy, and high rates of autism spectrum disorders and autistic features

Syndrome and/or gene affected	NMDA receptor effects	Presence of epilepsy	Presence of autism	Comments
Rett syndrome	NMDA antagonists ameliorate symptoms in mouse models [143, 144] and humans [143, 145]	Seizures common [146, 147]	ASD common [148, 149]	
Pitt-Hopkins syndrome	NMDA antagonists ameliorate symptoms in mouse models [150]	Seizures common [151]	ASD common [151, 152]	Amiable demeanor
Mowat-Wilson-syndrome	Increased NMDA activation in mouse model [153]	Seizures common [154]	ASD traits high but not higher than Intellectual Disability comparison group; high rates of repetitive behavior [155]	Amiable demeanor
Phelan-McDermid syndrome	Increased NMDA activation in mouse model of IB2 gene deletion [156]	Seizures common [157, 158]	Autism and autistic features common [156, 157, 159]	
CDKL5	Increased NMDA activation in mouse model [160]	Seizures common [160, 161]	Autistic features common [162–164]	Similar to Rett syndrome
MEF2C	Increased E/I ratio in mouse model, alleviated by NMDA antagonism [165, 166]	Seizures common [166, 167]	Autism and autistic features common [166, 168]	
Angelman syndrome	Decreased NMDA activation in mouse model [169]; higher E/I ratios and seizures due to reduced GABA-ergic activity [170]	Seizures common [171]	Autism common [172]	Happy disposition

For some of the disorders, NMDA antagonists (which cause psychotic symptoms in typical individuals) have also been shown to ameliorate these symptoms. These findings indicate that therapy with drugs that promote psychosis can, for some disorders, reduce symptoms of autism.

**Table 2.** Overview of main neurological and pharmacological systems discussed here, with reference to autism and psychotic-affective disorders

Pharmacological agent, system or therapy	Functions	Relevance in autism spectrum disorders	Relevance in psychotic-affective spectrum disorders	Implications for research and treatment
Brain derived neurotrophic factor	Mediates neurodevelopment, learning and memory, via growth and other effects	Elevated compared with controls on average	Reduced compared with controls on average	Test levels; modify receptor activation pharmacologically
PI3K-Akt-mTOR pathway	Mediates cell growth and replication across many tissues, including brain	Higher activity compared with controls on average	Lower activity compared with controls on average, for schizophrenia	Quantify activity levels; modify pharmacologically; effects systematic across body
Kynurenine pathway	Controls metabolism of tryptophan; affects NMDA receptor activity	Some evidence that kynurenic acid level is lower, quinolinic acid level is higher, on average	Some evidence that kynurenic acid level is higher, quinolinic acid level is lower, on average, in schizophrenia	More data needed; test levels of metabolites; relate to NMDA activity, EEG excitation/inhibition ratios
NMDA receptor antagonism and agonism	Major neurotransmission system with diverse effects on cognition, learning and memory; mediates seizures	Elevated NMDA activity and seizures in many cases of autism	Psychosis caused by blockade of NMDA receptor; hypofunction may also degrade cognition	More data needed on NMDA activity and function in idiopathic autism; negative allosteric
	(activation too high) or psychosis (activation blocked)			modulation of NMDA receptors may be useful in some cases of autism
Agmatine and arginine pathway	Agmatine acts as neurotransmitter that antagonizes NMDA receptor	Lower levels of agmatine in autism, on average	Higher levels of agmatine in schizophrenia and psychosis, on average	More data needed; test levels; diet can alter levels in blood
Endocannabinoid pathway; levels of anandamide	Anandamide mediates social behavior and many other physiological and neurological phenotypes	Lower levels of anandamide in autism, on average	Higher levels of anandamide in schizophrenia, on average	More data needed; test levels; modify pharmacologically
MDMA ('ecstasy')	Modulates serotoninergic, dopaminergic and norepinephrinergic neurotransmission	Data suggests enhancement of empathy and reduction in social anxiety; possible benefits in autism	Anecdotal data suggests may precipitatepsychotic episodes, in vulnerable individuals	More data needed; may be useful in autism in conjunction with psychological therapy
Antipsychotic therapy involving or eliciting autism traits	Antipsychotic drugs increase risk of seizures; electroconvulsive therapy induces seizures	Seizures and high excitation/inhibition ratios common in autism	Seizures may make individuals with schizophrenia or depression ‘more autistic’, neurochemically	Determine neurochemical mechanisms of electroconvulsive treatment
Prenatal and environmental drugs	Drugs given in pregnancy, or found in environment; are evolutionarily novel	Prenatal antidepressants and antipsychotics may slightly increase relative risk of autism	Any drugs developed to alleviate autism may have propsychotic effects prenatally	More data needed

See text for details and citations.

In contrast to the largely negative results described above, the mild NMDA antagonist memantine has shown effectiveness in improving sociality and reducing repetitive behavior among children with idiopathic autism [180–182]. A hyperglutamatergic model for idiopathic autism was originally described by Fatemi [183], and recent evidence salient to this theory was described in Rojas [184], including, e.g. high glutamate levels in brain tissue and blood (e.g. [185]), and therapeutic benefits from the NMDA receptor antagonists amantadine and acamprosate, in small trials.

Mouse autism models based on gene knockouts that impact glutamatergic activity (and on prenatal valproic acid effects), show increases in NMDA and/or mGlur5 activity that are reduced by antagonists and negative allosteric modulators, with consequent reductions in social deficits, repetitive behavior or both; such effects have been demonstrated for the genes ADCY5 [186], EiF4ebp2 [187], FMR1 [188], IRSp53 [189], Shank2 [190], TSC1 [191], as well as for 16p11.2 deletions [192] and prenatal valproate [193]. The high diversity of autism mouse models involved in these convergent studies indicate that hyper-glutamatergic neuronal activity characterizes a considerable subset of autism genes, and perhaps also a high proportion of subjects with idiopathic autism. As for Fragile X syndrome, efficacy of glutamate system antagonists in translation to humans remains to be demonstrated; consideration of what components of these systems differ, neurophysiologically, between mice and humans, and in autism compared with psychosis, should be useful in progress toward effective therapies.

Considerable work on the pharmacotherapy for schizophrenia centers on enhancing NMDA function, either through positive allosteric modulation of the receptor, or by enhancement of mGlur5 activation with secondary positive effects on NMDA activity [194–196]. Such studies have shown antipsychotic and pro-cognitive effects in some animal models, although side effects limit their effectiveness [197]. Positive allosteric modulation of the glycine-binding site of the NMDA receptor has demonstrated effectiveness, in animal models of schizophrenia, for improving cognition [198, 199]; conversely, antagonism of this site has been suggested as a therapy for autism [200]. Uno and Coyle [201] discuss the current status of these and other approaches to upregulating glutamatergic neurotransmission in schizophrenia. Such studies are especially important because most antipsychotic drugs work by down-regulating the dopaminergic activity that drives hallucinations and delusions, while not impacting upon cognitive-deficit symptoms that stem, in part, from reductions in the NMDA receptor activity required for effective synaptic plasticity.

### Agmatine and the arginine pathway

Agmatine is a compound, formed naturally by the decarboxylation of the amino acid arginine, that functions as a neurotransmitter. It acts as a ligand for the NMDA receptor, α2 adrenergic receptors, imidazoline receptors and neuronal nitrous acid synthase, being released by Ca++ dependent depolarization [202, 203]. Agmatine helps to protects cells from glutamate excitotoxicity, and reduces the incidence and intensity of seizures [202], apparently through its antagonism of the NMDA receptor [203, 204].

Substantially and significantly higher plasma agmatine has been reported in schizophrenia or first-episode psychosis subjects than in controls, by the three studies conducted to date [205–207], and treatment with antipsychotics significantly reduces its levels [207]. In a rodent model, high doses of agmatine cause disrupted prepulse inhibition, a strong phenotype of schizophrenia [208]. This set of findings is concordant with propsychotic effects of agmatine due to antagonism of the NMDA receptor.

Significantly lower plasma levels of agmatine were reported among autism subjects than controls in the single study of autism conducted thus far [209]. Agmatine treatment also rescues autistic behavior in the VPA mouse model of autism, in a manner comparable to that of two of the other NMDA antagonists, MK-801 and memantine [210], all of which alleviate social deficits associated with an overactive NMDA receptor [193]. Such treatments may thus ‘push’ individuals in the direction of psychosis, but only so far as to more or less normalize their cognition.

These findings on agmatine in relation to autism and schizophrenia or psychosis provide support for diametric pharmacological effects of this compound in these two disorders, involving low versus high plasma levels, and pro-psychotic, anti-autistic effects mediated by NMDA antagonism. Further studies are needed to determine the generality of the patterns, preferably through analyses of autistic and psychotic subjects using the same protocols.

### Endocannabinoid system

The human endocannabinoid system comprises two endocannabinoids that serve as neurotransmitters (anandamide and 2-arachidonoylglycerol), the enzyme system that synthesizes and degrades them, and the cannabinoid receptors CB1 and CB2. This system exhibits a wide range of physiological and neurological functions, including effects on social behavior, the oxytocin system and NMDA receptor activation [211–213]. Anandamide in particular is known to mediate variation in social behavior, such that higher levels are linked with increased social approach, social interaction, social play and social reward, from diverse studies of rodents [214].

Levels of anandamide in blood and cerebrospinal fluid are significantly higher among individuals with schizophrenia, compared with controls, by meta-analysis [215]. In contrast, blood (serum or plasma) anandamide levels are significantly lower, compared with controls, among individuals with autism in the two studies conducted to date [216, 217]. Blockade of the enzyme that degrades anandamide, leading to higher levels, also reverses social impairments found in two different autism mouse models, BTBR and *fmr1* knockouts [218]. The mechanisms involved in the differences between autism and schizophrenia remain unclear, although they may, as noted above, involve oxytocin signaling or modulation of NMDA receptor activation.

The exogenous cannabinoids Δ-9-tetrahydocannabidiol (THC) and cannadibiol (CBD), derived from the cannabis plant, exert complex effects on the human endocannabinoid system and relevant aspects of cognition, all of which are evolutionarily novel and thus challenging to interpret as regards adaptive function in humans. THC has been reported to increase sociality and empathy in humans, from self-report studies, as well as inducing, in high doses and susceptible individuals, symptoms of psychosis (reviews in [214, 219]). In contrast, CBD appears to exert antipsychotic effects via mechanisms that remain unclear [220].

Future work on the human endocannabinoid system might usefully focus on pharmacological means to modulate levels of anandamide, and on the mechanisms whereby CBD reduces symptoms of psychosis. This system also highlights the distinction between pharmacological modulation of pathways with endogenous bodily neurochemicals (such as anandamide), compared with evolutionarily novel ones produced by plants or in the laboratory (such as THC, CBD and their synthetic derivatives). In principle, endogenous neurochemicals should less likely to exert deleterious side effects, as they are natural components of an adaptive, evolved neurological system.

### MDMA

MDMA (‘ecstasy’) modulates monoaminergic neurotransmission especially with regard to serotonin, dopamine and norepinephrine availability at synapses. Its use enhances empathy and prosocial behavior, and involves increased plasma oxytocin [221–224]. These properties of MDMA have been applied successfully in reduction of social anxiety among autistic adults [225], and should also be effective in increasing levels of empathy and sociality among individuals with autism [226]. Current clinical use focuses on MDMA-assisted psychotherapy for post-traumatic stress disorder, for which it is in phase 3 trials as of early 2019 [227].

Under the supposition that MDMA may serve as a useful treatment for autism due to its prosocial effects, it would be predicted to increase psychosis-related phenotypes. Numerous reports describe psychosis and psychotic episodes associated with MDMA use [228–232], and Duman *et al.* [233] reported that MDMA and cannabis use interact additively in elevation of subclinical psychotic trait scores. However, given that most MDMA users commonly use various other psychoactive drugs, it remains unclear to what degree MDMA in particular is involved in these cases and effects [234].

### Antipsychotic treatments eliciting autism-related traits

The diametric theory for autism and psychosis predicts that antipsychotic treatments should exert effects that direct neurological and psychological systems in the direction of autistic phenotypes. As such, antipsychotic treatments should both normalize aspects of cognition and, if their effects are stronger, generate autism-related traits. Evidence regarding such effects comes from four main lines of evidence: (i) the induction of seizures by antipsychotic drugs among individuals with psychosis; (ii) the induction of psychosis by anticonvulsant (antiepileptic) drugs among individuals with epilepsy; (iii) the success of electroconvulsive therapy (ECT) for psychosis and depression; and (iv) the induction of autism-related traits, in the form of obsessive-compulsive behaviors by antipsychotic drugs. The former line of evidence are predicated on the high comorbidity of idiopathic autism with epilepsy noted above, such that coincidence rates range from ∼15 to 50% [24–28]. High rates of epilepsy are also found in syndromic forms of autism, as shown, e.g. in Table 1, and high rates of subclinical autistic traits are reported among individuals with epilepsy [26, 235].

Antipsychotic drugs are known to induce seizures in a dose-dependent manner, as well as causing epileptiform EEGs [236–238]. The mechanisms of such effects remain unclear, but they may involve alterations to dopaminergic, cholinergic or inhibitory GABAergic neurotransmission by antipsychotics; reductions in antipsychotic-induced seizures especially by valproic acid, which elevates levels of GABA, suggest that this neurotransmitter, which works mainly in opposition to the excitatory effects of glutamate, plays an important role [239]. From the diametric theory evaluated here, antipsychotic drugs reduce the threshold for seizures in the context of elevating ratios of excitatory to inhibitory neurotransmission, especially involving the NMDA receptor, generating this autism-associated trait.

The converse to antipsychotic induction of epilepsy among subjects with psychosis, the induction of psychosis by anticonvulsants among individuals with epilepsy, also represents a well-known phenomenon [240], especially in the context of the so-called ‘forced normalization’. This term was coined in the 1950s for the association of psychosis onset with normalization of EEG findings, such that epilepsy and psychosis exhibit an inverse, antagonistic relationship [241–243]. Krishnamoorthy *et al.* [241] suggested that forced normalization was caused by changes to NMDA receptor activation, from high (associated with epilepsy) to low (associated with psychosis), with changes to this receptor potentiated by drug-induced GABAergic alterations; they also note that ‘anticonvulsants that increase GABA levels are associated with a psychopathological state in up to 10% of patients, characterized by mood changes, agitation, and paranoid psychotic symptoms’. Consideration of forced normalization in the context of the diametrical theory may help to clarify its causes and identify individualized risk factors and levels associated with position along a spectrum from autism, to normality, to psychosis.

ECT treatment, an effective therapy for otherwise treatment-resistant schizophrenia and severe depression despite its controversial nature [244], represents artificial induction of generalized seizures. As such, it can be considered as imposition of an autism-related brain activation state, with consequent changes in brain gene expression and neurological phenotypes that are predicted, by the theory addressed here, to be similar to those found in autism. Rosenquist *et al.* [245] noted that, given the ameliorative effects of ECT on psychotic states that are caused by NMDA blockade (with PCP, amphetamine, or other agents), as well as idiopathic and schizophrenia-associated psychosis, this treatment can be regarded as effective against psychosis in particular rather than schizophrenia in general. ECT apparently exerts its effects at least in part through changes in the glutamate-GABA system; in depression, beneficial effects also appear to be associated with normalization of glutamatergic neurotransmission, from studies of rodents [246, 247]. In both schizophrenia and depression, the mechanism of ECT involves increased levels of BDNF [49, 248–250], as found typically in subjects with autism as described above. These findings as regards effects on the glutamatergic system, and BDNF increases, are also supported by changes in gene expression following electroconvulsive treatment in rats [251]. Taken together, these diverse results are consistent with autism-related alterations to brain neurochemistry and synaptic activity following ECT; analyses that test directly for such effects, across many genes, would provide further evaluation of this hypothesis.

Finally, one of the most curious and puzzling side effects of some antipsychotics is the emergence after treatment of symptoms of obsessive compulsive behaviors and disorder, among patients with schizophrenia [252, 253]. This phenomenon is of interest given the strong overlap of autism and autism-related traits with obsessive compulsive behaviors and disorder [254–256], and the fact that OCD-related behaviors commonly include ordering, extreme neatness, counting and repetitive actions that are characteristic of the autism spectrum. As described by the sociologist Joan Donovan, from when she worked in group homes for patients with mental illness:

Many of the psychiatrists in the area were fond of prescribing clozaril/clozapine, a “drug of last resort.” As counselors, we were told a lot about this drug because the side effects (reduced white blood cells) could be fatal.

As more clients began taking it, I noticed some consistent changes in their behavior. They became far more interested in making sure they were taking their meds regularly, would attend day programs, appointments, or work more frequently. As well, they would spend lots of time organizing their rooms. Counselors were also responsible for doing periodic checks for cleanliness and hazards. Clozaril patients often prepared for the inspection with great care: lining books on shelves by size and color, stacking food in the pantry or fridge in order of size, hanging their clothing or putting it in drawers sorted by type. Some became very focused on one or two niche subjects (art, music, books, film) and would seek out and collect these items by a single artist or author. As you can imagine, watching someone with schizophrenia undergo this transformation was promising, especially because the hallucinations disappeared along with the ego.

Over time though, the side effects of clozaril took their toll … and some could no longer tolerate it. Once off the meds, they returned to their normal disorganization and isolation.

Might such effects emerge, in part, as a result of autistic-trait induction?

### Prenatal and environmental drugs affecting risks of autism and psychotic-affective disorders

By the hypothesis evaluated here, antipsychotic and antidepressant drugs are expected to direct cognition toward autism spectrum phenotypes, during both postnatal stages and prenatal development. Prenatal effects are especially likely as regards autism risk, given that this condition is typically present from birth and centrally involves altered early neurodevelopment.

Hadjikhani [257] suggested that SSRI use during pregnancy mediates increased risk of autism, based especially on well-replicated evidence of high platelet serotonin in autistic children. Recent meta-analyses have supported this supposition, with ∼2-fold higher rates of autism among children born to mothers who took SSRIs during pregnancy [258, 259]. Prenatal SSRI use influences aspects of human brain structural development [260] and language acquisition [261], but the mechanisms of apparent SSRI impacts on autism risk remain unclear.

Effects of prenatal antipsychotic use during pregnancy on autism risk are largely unknown, due to a paucity of relevant quality data [262, 263]; one study [264] reported increased head circumference, an autism-associated trait, in neonates prenatally exposed to the antipsychotics olanzapine and/or clozapine, but birth weight and length, which are also commonly higher in autism, were not affected.

Valproic acid, which is prescribed for mania in bipolar disorder, as an adjunctive treatment (secondary, along with antipsychotics) in schizophrenia, as well as for epilepsy, is a well-established risk factor for autism when given prenatally, and indeed it has been developed into an animal model of autism in this context [46, 265, 266]. Prenatal valproic acid induces local cortical hyperconnectivity and hyper-neuronal excitability, both strong neural correlates of autism [267, 268]. In adults, valproate treatment also ‘reopens’ a developmental critical period for the development of absolute pitch [269], another autism-enhanced trait (review in [270]) and one that shows major deficits in subjects with schizophrenia [271].

Prenatal effects on risk of autism and other disorders can also be mediated by pharmacological agents present environmentally, as in drinking water, given that many such drugs persist in aquatic systems [272]. As evidence of such effects, fathead minnows exposed to high but environmentally-realistic concentrations of the drugs fluoxetine, venlafaxine and carbamazepine showed gene-expression changes that were uniquely enriched for autism risk genes [273, 274]. The potential for impacts on humans of drinking water pharmacological exposures is indicated by studies showing inverse associations of natural geographic variation in levels of lithium in drinking water with rates of depressive and psychotic symptoms [275, 276].

## DISCUSSION

I have described and evaluated a set of hypotheses predicated on a diametric structure to autism spectrum versus psychotic-affective spectrum conditions, which predicts that pharmacological treatments for each set of disorders should likewise be diametric. A large set of diametric psychopharmacological patterns emerged from the analyses, with regard to the pathways involving BDNF, PI3K, the NMDA and mGlur5 receptors, kynurenine, agmatine, the endocannabinoid anandamide, epilepsy and electroconvulsive therapy. The main results, and consequent suggestions for future work, are summarized in Table 2. These findings suggest new avenues for therapy in both autism and psychotic-affective conditions, provided that differential diagnoses based on the relevant neurochemical, pathway or ligand/receptor system indicate that it is warranted in any particular subject. The hypothesis evaluated here also makes extensive predictions regarding neurochemical levels and pathway activation patterns in autism compared with psychotic-affective conditions, in that such levels and patterns should commonly show diametric, opposite effects. As such, this work can also help to guide future data collection on other psychopharmacological systems, such as amphetamines and other stimulants (e.g. methylphenidate), which are commonly prescribed for autism [277] and can induce psychosis (e.g. [278]). Most generally, a useful finding from autism regarding some effect will thus immediately suggest that the same phenotype be investigated in, say, schizophrenia and vice versa.

The primary limitations of this study include: (i) the incomplete amount of relevant information available for some disorders, for some neurochemicals, such that further data, and meta-analyses, are required for robust inference; (ii) the fact that very few studies have directly compared autism with schizophrenia or other psychotic-affective disorders, with regard to neurochemicals, pathway activation, or psychopharmacological effects, (iii) the high etiological and phenotypic heterogeneities of the disorders involved, which means that any given case of autism, or schizophrenia, may have quite different causes than another, and (iv) the presence of contradictory evidence, such as anticonvulsant use for some psychotic-affective disorders, antipsychotic use for some symptoms of autism (such as irritability and aggression) [21], and a negative genetic correlation of autism with localized epilepsy [279]. The former two limitations can be alleviated by targeting data collection toward key systems and neurochemicals, as noted above and in Table 2. Effects of the third limitation can be reduced through the realization that mental disorders require personalized treatments with regard to psychopharmacology and other therapeutic approaches, that can only be realized through development of protocols for systematic differential diagnoses of causes rather than just disorders themselves. This article serves as a first step toward this goal, by demonstrating the wide range of etiological factors that may be involved, in the context of reciprocal illumination between autism and psychotic-affective disorders. Finally, this article does not consider structural aspects of brain development, which are mediated by neurochemicals but in ways that are not sufficiently understood as yet for meaningful comparisons of autism with psychotic-affective disorders.

In the context of therapy, diametric effects on autism and psychotic-affective disorder etiologies also extend to protection from disorders in high risk individuals, given that some correlates of autism, e.g. effects of high birth weight [280], effects of the dup22q11.2 CNV [281], effects of congenital blindness [282, 283], and effects of a high non-verbal relative to verbal IQ [284] are known to protect against schizophrenia; might more malleable autism-associated factors, such as enhanced sensory focus and abilities, high spatial abilities such as on the embedded figures test, low imagination and highly focused attention exert similar protection among individuals at high risk of schizophrenia? By similar mechanisms, schizophrenia symptoms could also be alleviated by targeted enhancement of ‘autistic’ traits, such as high sensory abilities. For example, training of people with schizophrenia to enhance sensory acuities, which are generally higher in autism but reduced in schizophrenia (see [285]), has been shown to increase both sensory skills and aspects of cognition [286].

Conversely to these effects, prevention or alleviation of autistic traits may usefully focus on enhancing imagination [287], reducing extreme attentional focus [288], promoting metaphoric verbal skills over non-verbal abilities, fostering reduction of sensory acuity focus, and in general promoting interventions that direct phenotypes in the general ‘direction’ of psychosis, but with a target zone in the typical cognition range. As for pharmacological treatments, such approaches must be highly personalized to each individual's neurological, psychological and physiological makeup, through extensive testing prior to treatment strategy development. Doing so requires a fundamental change in psychiatric mind-set, because designations of autism, or schizophrenia, become, under this paradigm, only starting points for differential diagnosis of their highly individualized causes and treatments.

**Conflict of interest:** None declared.
